# Mitochondrial Genome Reduction and Accelerated Evolution in Planktonic Foraminiferans

**DOI:** 10.1002/mbo3.70368

**Published:** 2026-07-30

**Authors:** Hsin‐Tung Lai, Ming‐Wei Lai, Tzu‐Haw Wang, Haojia Ren, Chuan Ku

**Affiliations:** ^1^ Institute of Plant and Microbial Biology Academia Sinica Taipei Taiwan; ^2^ Institute of Ecology and Evolutionary Biology National Taiwan University Taipei Taiwan; ^3^ Department of Geosciences National Taiwan University Taipei Taiwan

**Keywords:** comparative genomics, Foraminifera, mitogenome, protist, reductive evolution, Rhizaria, single‐cell genomics

## Abstract

The evolution of mitochondria provides crucial insights into the diversification of eukaryotes, with complex events of gene losses revealed through comparative analyses of mitochondrial genomes (mitogenomes) across eukaryotic lineages. However, the mitogenomes of many microbial eukaryotes remain underexplored due to challenges in their isolation and cultivation. Particularly understudied are Foraminifera (Rhizaria, SAR), unicellular calcifiers that are widely distributed across global oceans and important paleoenvironmental proxies. Using single‐cell genomic sequencing, we report a 22‐kb mitogenome from a planktonic foraminiferan in tropical seawater, the smallest known to date among sequenced mitogenomes of Rhizaria, a major lineage of eukaryotes. It contains only six protein‐coding genes (including reduced versions of *nad1* and *cox2*) and fragmented ribosomal RNA genes, and has lost most genes in oxidative phosphorylation and all genes encoding mitochondrial ribosomal proteins. Such genome reduction is associated with accelerated evolutionary rates and a lower GC content than that of benthic foraminiferan and other rhizarian mitogenomes. These findings highlight a unique trajectory of mitogenome reduction during rhizarian evolution and the use of single‐cell approaches to recover microbial eukaryotic genomes and expand our understanding of mitochondrial evolution.

## Graphical abstract

The mitochondrial genomes of the eukaryotic lineage Retaria (Foraminifera and Radiolaria) show reductions in multiple gene categories, including complex I, transfer RNAs (tRNAs), ribosomal proteins, and ribosomal RNAs. In planktonic foraminifera, this reduction is even more pronounced, with the potential loss of *atp* and other genes.

## 1 Introduction

Mitochondria are endosymbiotic organelles that originated from free‐living alphaproteobacteria (Zimorski et al. 2014; Archibald 2015). Over time, mitochondrial genomes have undergone gene loss and endosymbiotic gene transfer to the nuclear genome, resulting in extensive gene reduction and diversification of mitogenome functions and structures (Timmis et al. 2004; Ku et al. 2015; Janouškovec et al. 2017; Butenko et al. 2024). Animals and plants have more than 20,000 complete mitogenome sequences in the NCBI Organelle Database (released on February 12, 2025). In contrast, many microbial eukaryote taxa, particularly those that cannot be easily cultured, have few mitogenome sequence records (Zardoya 2020), although their mitogenomes demonstrate greater diversity (Smith and Keeling 2015). It is thus necessary to obtain a more extensive sampling of microbial eukaryotes to better understand mitogenome evolution.

As the primary site of energy production, mitochondria typically retain genes encoding proteins involved in oxidative phosphorylation (electron transport chain [ETC; complexes I–IV] and ATP synthase [complex V]) and translation (ribosomal proteins, rRNA, and transfer RNA [tRNA]) (Gray 2014). Mitogenomes show remarkable variation in gene contents, with up to 66 protein‐coding genes in *Andalucia godoyi* (Discoba) (Burger et al. 2013). Different degrees of mitogenome reduction are observed across the tree of eukaryotes, with complete loss of mitochondrial DNA in diverse anaerobes that have the derived forms of mitochondria, hydrogenosomes, or mitosomes (Müller et al. 2012). In the facultative anaerobic alga *Euglena gracilis* (Discoba), which can use fumarate as a terminal electron acceptor during anoxia, the mitogenome has lost several complex I genes and its rRNA genes are fragmented (Müller et al. 2012; Dobáková et al. 2015). In diverse myzozoans (Alveolata), including apicomplexan parasites, dinoflagellates, and *Chromera*, mitochondria generally have fewer than five protein‐coding genes and their ribosomes contain dozens of small rRNA fragments (Waller and Jackson 2009; Flegontov et al. 2015; Shikha et al. 2025).

Rhizaria, which comprises Cercozoa, Endomyxa, and Retaria (Adl et al. 2019), are diverse free‐living or parasitic, ameboid eukaryotes and one of the least studied eukaryotic supergroups in terms of mitogenomes, with only 12 reference mitogenome sequences (four cercozoans, four endomyxans, and four retarians) currently available. In Retaria, Foraminifera are marine, mostly calcifying, large unicellular eukaryotes, living either as free‐floating planktonic or bottom‐dwelling benthic organisms. Due to their wide distribution and preservable calcareous shells, foraminiferans play crucial roles in carbon cycling and serve as important bioindicators and microfossils in paleoceanographic studies (Kucera 2007; Yasuhara et al. 2017).

Available genomic data for Foraminifera are mostly limited to specific gene fragments (e.g. nuclear small‐subunit [SSU] ribosomal DNA [rDNA], Pawlowski et al. 2013; Morard et al. 2024; mitochondrial *cox1* gene, Macher et al. 2022) obtained from environmental samples. Whole‐genome sequencing data exist for only three benthic species: *Astrammina rara* (Habura et al. 2011), *Globobulimina* sp. (Woehle et al. 2018), and *Reticulomyxa filosa* (Glöckner et al. 2014). In addition, circularized mitogenome sequences have been reported from two benthic foraminiferans, *Neorotalia gaimardi* and *Calcarina hispida*, which have reduced gene contents that lack all of the ribosomal protein genes (Macher et al. 2023). To date, no whole‐genome sequencing data are currently available for planktonic foraminifera, despite their ecological significance and global fossil records.

In this study, we applied a single‐cell (single‐particle) approach to genome sequencing for planktonic foraminifera. This culture‐independent method enables the examination of sequence information from individual cells and has important potential for the study of microbial eukaryotes (Ku and Sebé‐Pedrós 2019; Ciobanu et al. 2021; Keeling 2019; Schön et al. 2021). Although the coverage of nuclear genomes has been limited by the low‐copy nature of the nuclear DNA molecules, single‐cell genomics can generate complete genome sequences of the mitochondria (Macher et al. 2023; Wideman et al. 2019; Záhonová et al. 2021), which can have hundreds to thousands of copies within a cell (Mohamed Yusoff et al. 2025). Using this approach, we recovered a complete, circularized mitogenome sequence of a planktonic foraminiferan isolated from tropical seawater and conducted comparative and phylogenomic analyses of foraminiferan mitogenomes.

## 2 Materials and Methods

### 2.1 Sample Collection

Marine particles were collected in August 2023 by towing a 200‐µm‐mesh plankton net at a depth of ~13 m near Gong‐Guan Harbor, Green Island, Taitung County, Taiwan (22.69° N, 121.48° E), where planktonic foraminiferans have been observed (Cai‐Li et al. 2025; Fang et al. 2025). COPAS Vision, a large‐particle imaging flow cytometer (Union Biometrica, Holliston, USA), was used for fast preliminary examination of particle composition. To preserve the integrity of foraminiferan particles, we manually isolated individual particles with a micropipette and observed them under a Nikon ECLIPSE Ts2‐FL inverted optical microscope (Nikon, Tokyo, Japan). The particles were washed with artificial seawater, individually transferred into a 96‐well plate with each well containing 100 µL 5% dimethyl sulfoxide in artificial seawater, and preserved at −20°C until further processing for DNA extraction.

### 2.2 Sample Preparation for Scanning Electron Microscope (SEM) Observation

For SEM observation, 40 mL of the net tow material was fixed with 1% formaldehyde and 0.05% glutaraldehyde to preserve microbial morphology. The sample was filtered onto a 0.2‐µm polycarbonate membrane and rinsed with phosphate buffer, followed by ethanol dehydration and critical point drying (EM CPD300; Leica, Wetzlar, Germany). Finally, it was cut into sections and mounted onto stubs for SEM imaging (FEI Quanta 200; FEI, Hillsboro, USA).

### 2.3 DNA Extraction and Sequencing

Single foraminiferan particles were individually transferred to 1.5‐mL Eppendorf tubes and ground into powder using a customized plastic stick with a granular head that fits the bottom of the Eppendorf tube. Cell lysis and whole‐genome amplification were carried out using the REPLI‐g Advanced DNA Single‐Cell Kit (QIAGEN, Hilden, Germany) following the manufacturer's protocol. The amplified DNA fragments were purified using the AMPure XP Bead‐Based Reagent (Beckman Coulter Life Science, Brea, USA). Purified DNA samples were quantified using Qubit HS (Thermo Fisher Scientific, Waltham, USA). Libraries were prepared using Illumina DNA Prep (Illumina, San Diego, USA), with the lengths estimated by Fragment Analyzer (Agilent, Santa Clara, USA), and sequenced on the Illumina NovaSeq. 6000 platform (150‐bp paired‐end reads). The remaining DNA samples of three particles (GI‐A, GI‐C, and GI‐D) were also sequenced using a Nanopore R10.4.1 flowcell (Oxford Nanopore Technologies, Oxford, UK).

### 2.4 Genome Assembly

Illumina short reads were processed with fastp v0.22.0 (Chen et al. 2018) for adapter removal and trimming. For the Nanopore long reads, real‐time basecalling was performed using MinION MK1C (Oxford Nanopore Technologies, Oxford), and the reads were trimmed using Porechop v0.2.4 (Wick et al. 2018). Illumina short reads were assembled using SPAdes v4.0.0 (Prjibelski et al. 2020) in single‐cell mode (‐‐sc) with specified *k*‐mers (‐*k* 21, 33, 55, 77, 99, 127). Assembled contigs were then classified by Tiara v1.0.3 (Karlicki et al. 2022) into six groups based on *k*‐mer frequencies (Archaea, Bacteria, Prokarya, Eukarya, Plastid, and Mitochondrion). The contigs assigned to the group “Mitochondrion” were searched using the *blastx* command in BLAST+ v2.13.0 (Camacho et al. 2009) against a custom protein database containing protein‐coding sequences of the two available foraminiferan mitogenomes (benthic species *C. hispida* [OP965950] and *N. gaimardi* [OP965949], Macher et al. 2023) and representatives of Radiolaria (*Acanthometra* sp. and *Lithomelissa* sp., Macher et al. 2023) and Cercozoa (*Lotharella oceanica* [NC_029731], Tanifuji et al. 2016) using an *E* value cutoff of 1e ‐10. Similarly, we also searched the assembled contigs without the “Mitochondrion” label against the same custom database to find any additional contigs of mitochondrial origins. All the contigs with hits to this database were extracted and individually served as seed inputs to NOVOPlasty v4.3.1 (Dierckxsens et al. 2016).

### 2.5 Quality Assessment of Genome Assembly

Illumina and Nanopore reads were mapped to the best assembled mitogenome sequence (Globigerinidae sp. GI‐D) using Bowtie2 v2.4.1 (Langmead and Salzberg 2012) and Minimap2 v2.28‐r1209 (Li 2018), respectively, for visual inspection in Integrative Genomics Viewer (Thorvaldsdottir et al. 2013) to ensure the correctness of the genome assembly. The circularity and completeness of the mitogenome were confirmed by examining the continuity of both long and short reads and by shifting the endpoints of the assembly by 1 kb. The quality of the circularized mitogenome sequences was further evaluated by QUAST v5.0.2 (Gurevich et al. 2013). Repetitive sequence analysis of the mitogenome was conducted using PERF v0.4.6 (Avvaru et al. 2018).

### 2.6 Genome Annotation

The Globigerinidae sp. GI‐D mitogenome was annotated using the MFannot web server (Lang et al. 2023) and MITOS2 (Bernt et al. 2013) with reference genome set refseq. 63o. The genetic code was set to NCBI genetic code 4 (the mold, protozoan, and coelenterate mitochondrial code, and the mycoplasma/spiroplasma code) for annotating mitochondrial protein‐coding, tRNA, and rRNA genes. Unannotated regions were translated into amino acid sequences also using the NCBI genetic code 4 and searched against the predicted protein sequences from the two available foraminiferan mitogenomes (*C. hispida* and *N. gaimardi*, Macher et al. 2023) using *blastx*. The annotations were visualized using the OGDRAW web server (Greiner et al. 2019).

### 2.7 Phylogenetic Analyses

Amino acid sequences of whole mitogenome protein‐coding genes of Globigerinidae sp. GI‐D and other Rhizaria species, retrieved from NCBI and previous publications (Table S1), were aligned and concatenated using OrthoFinder v2.5.5 (Emms and Kelly 2019). A maximum likelihood phylogeny was inferred with IQ‐TREE v2.2.2.7 (Nguyen et al. 2015), using ModelFinder (Kalyaanamoorthy et al. 2017) to determine the best‐fit substitution model according to the Bayesian information criterion. The final model was selected manually, taking into account the intended use and assumptions underlying each model. For individual gene trees, sequence alignments generated with MAFFT v7.520 (Katoh and Standley 2013) and phylogenies were reconstructed using IQ‐TREE in the same way. Phylogenetic trees were visualized in FigTree v1.4.4 (Rambaut 2012).

## 3 Results

### 3.1 Complete Mitogenome Sequence From a Planktonic Foraminiferan

**Figure 1:**
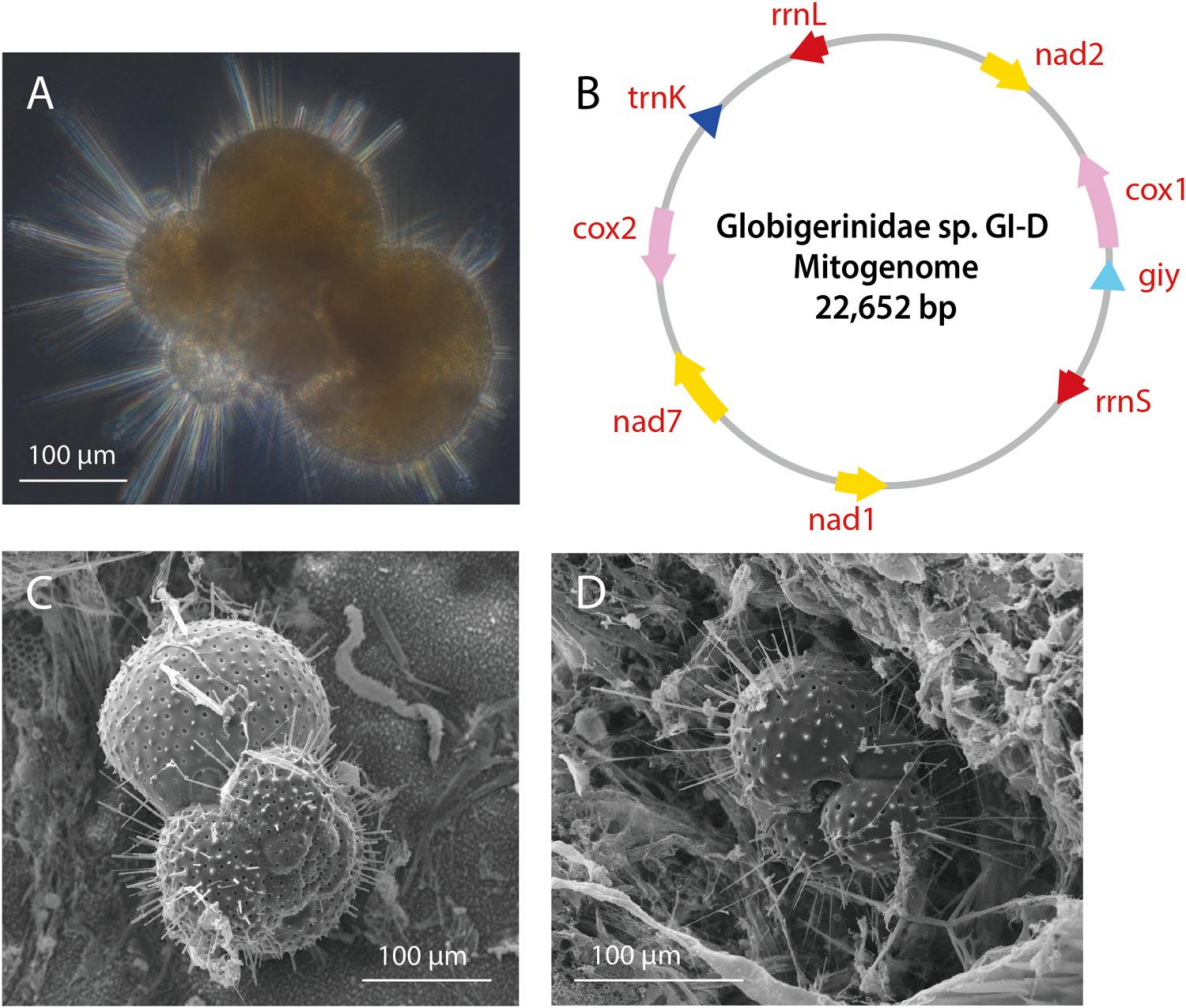
Globigerinidae and its mitochondrial genome sequence. (A) The planktonic foraminiferan particle GI‐D from the family Globigerinidae before DNA extraction. (B) Mitogenome map of GI‐D, showing genes in different functional categories: yellow (complex I), pink (complex IV), red (ribosomal RNA), dark blue (transfer RNA), and light blue (others). Arrows indicate the transcriptional direction of each gene. (C, D) Scanning electron microscope images of morphologically similar particles from Globigerinidae in the same seawater sample.

In total, 20 foraminiferan particles from the family Globigerinidae (Rotaliida, Globothalamea) were isolated, which were the dominant taxon of planktonic foraminiferans in seawater offshore Green Island, Taiwan. After single‐cell genome amplification, we obtained enough DNA for four particles and sequenced their DNA. Among them, the single amplified genome (SAG) from the particle Globigerinidae sp. GI‐D has the best assembly quality supported by both Illumina and Nanopore reads (Table 1). Although each particle was thoroughly washed with aseptic artificial seawater, putative prokaryotic contigs that likely came from closely associated or attached prokaryotic cells still comprised the majority of all contigs for each particle, with GI‐D having the highest proportion of eukaryotic contigs (Table S2). Contigs showing high similarity to mitochondrial protein‐coding sequences from the two benthic foraminiferans, *N. gaimardi* and *C. hispida*, were identified and used as seed sequences (baits) to assemble more complete mitochondrial genomes using NOVOPlasty. This approach successfully recovered a circularized mitogenome sequence of the particle GI‐D (GenBank accession PV558267), which is 22,652 bp in length and has a GC content of 21.5% (Figure 1). Mapping of both Illumina short reads (7435× coverage) and Nanopore long reads (560× coverage) confirmed the contiguity of read coverage and the correctness of the assembly, including the circularity of the mitogenome, which was double‐checked by randomly shifting the start position of the mitogenome sequence. In addition, a 30,272‐bp circularized mitogenome sequence was recovered from a haptophyte alga *Chrysochromulina* sp. (Prymnesiales, Haptista) associated with GI‐D (GenBank accession PV535690). Other than the contigs corresponding to these two circularized mitogenome sequences, we did not find any other high‐coverage SPAdes‐assembled contig from GI‐D that has typical mitogenome‐encoded genes. Partial mitogenome sequences were assembled from two other planktonic foraminiferan particles, GI‐A (2279 bp) and GI‐C (6068 bp), whose mitochondrial *cox1* sequences are closely related to that of GI‐D (Figure S1).

**Table 1:** Assembly statistics of the Globigerinidae sp. GI‐D single amplified genome (SAG).

	Globigerinidae sp. GI‐D SAG
Illumina read data (Mb)	20,828.54
Nanopore read data (Mb)	1464.86
# Assembled contigs > 1000 bp	30,626
Assembly size (Mb)	180.6
GC content (%)	43.96
N50	9014
L50	3102
Illumina read coverage	153×

The particle GI‐D is multilocular with distinct spines extending from the chamber walls (Figure 1). Its globular chambers are arranged in a trochospiral coiling pattern, with four chambers clearly visible in the final whorl and smaller chambers coiled centrally. These morphological features strongly suggest that GI‐D is affiliated with the family Globigerinidae, as Globigerinidae spp. are spinose and exhibit trochospiral or near‐planispiral chamber arrangements (Carpenter et al. 1862). The chamber wall surface of GI‐D is rough and perforated, with a yellow–green color, which is likely due to symbiotic microalgae. Because only the lateral view of GI‐D was photographed, the aperture (the opening to the exterior on the final chamber) was unobservable, making it difficult to classify GI‐D to the species level.

### 3.2 Substantial Mitogenome Reduction and Gene Losses

**Figure 2:**
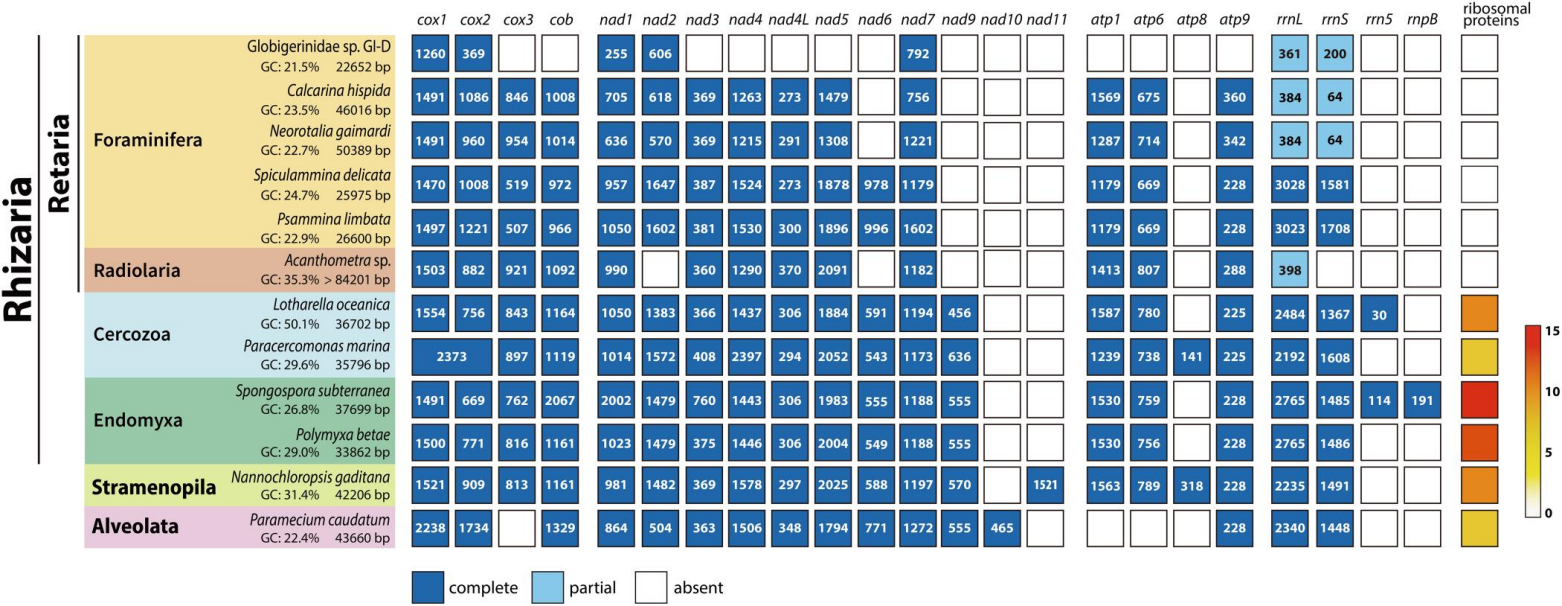
Comparative mitochondrial genomics of Rhizaria. The presence, partial presence, and absence of genes in mitogenomes from Rhizaria and representatives of Stramenopila and Alveolata are indicated by dark blue, light blue, and white blocks, respectively. Numbers within dark or light blue blocks represent gene lengths in bp. For ribosomal proteins, a red‐to‐white gradient indicates the number of ribosomal proteins encoded in each mitogenome.

The mitogenome of Globigerinidae sp. GI‐D is by far the smallest mitogenome among all rhizarian mitogenomes and contains only six protein‐coding genes and a single tRNA gene (trnK) (Figure 1). Similar to other retarians (except for the newly included xenophyophores *Spiculammina delicata* and *Psammina* aff. *limbata*), the GI‐D mitogenome only has fragmented large‐ and small‐subunit rRNA genes, in contrast to the full‐length rRNA genes found in cercozoans, stramenopiles, and alveolates (Figure 2). The retarian (Foraminifera and Radiolaria) mitogenomes have substantially reduced gene contents, characterized by the loss of *nad9*, *atp8*, and all ribosomal protein genes (Figure 2). The proportion of gene‐coding regions in globothalamid foraminiferans (17.4~33.5%) is significantly lower than in other rhizarian mitogenomes (e.g. *L. oceanica*, 95%; *Spongospora subterranea*, 66%), and the human mitogenome (93%, Chinnery and Hudson 2013) (Table 2).

**Table 2:** Mitogenome information of foraminiferan species (Globothalamea).

	Mitogenome size (bp)	GC content (%)	Gene‐coding region (%)	Repetitive content (%)	Repeat frequency (kb)	Accession ID
GI‐D	22,652	21.5	23.3	3.6	3.62	PV558267
*Calcarina hispida*	46,016	23.5	17.4	1.4	1.15	OP965950
*Neorotalia gaimardi*	50,389	22.7	33.5	1.1	1.49	OP965949

The planktonic foraminiferan GI‐D has a mitogenome that is even more reduced in both genome size and gene content compared with the two benthic foraminiferans *C. hispida* and *N. gaimardi*. Specifically, *cox3*, *cob*, *nad3*, *nad4*, *nad4L*, *nad5*, and all *atp* subunit genes are absent. Although small fragments with similarity to *cox3*, *nad5*, and *atp1* could be detected through MITOS2, these fragments are all less than 50 bp. On the other hand, the Globigerinidae sp. GI‐D mitogenome is uniquely annotated with a partial *giy* gene (encoding GIY‐YIG protein) and *trnK*, which are absent in the benthic species. Additionally, GI‐D has a lower GC content and a higher proportion of repetitive sequences in the non‐coding regions (Table 2 and Figure S2).

### 3.3 Phylogenetics and Accelerated Evolution of Foraminiferan Mitogenomes

**Figure 3:**
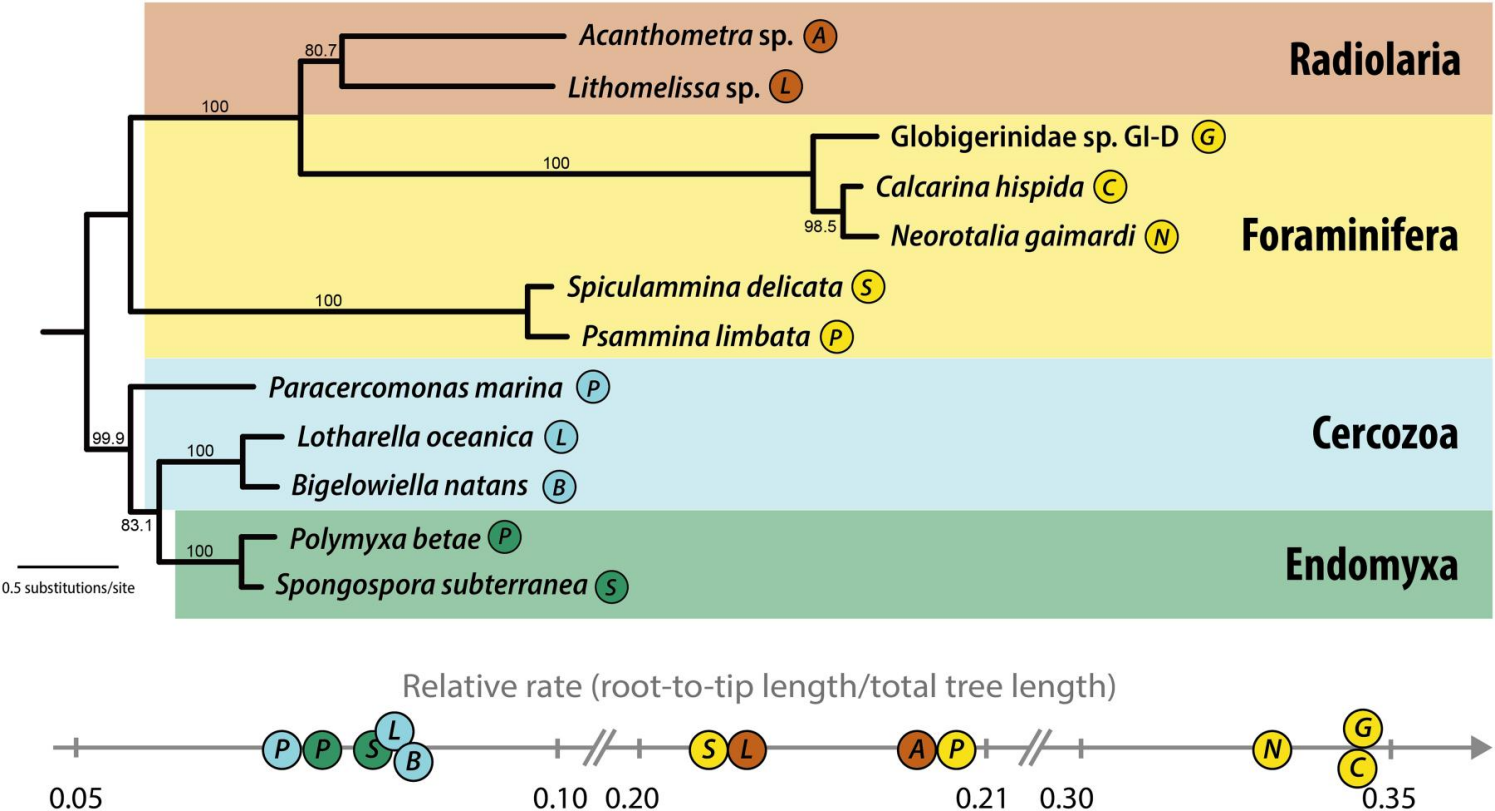
Whole‐mitogenome phylogeny of Rhizaria and their relative evolutionary rates. The maximum likelihood tree was constructed using protein sequences COX1, COX2, NAD1, and NAD7 predicted from whole mitogenomes and the mtInv + F + R4 model implemented in IQ‐TREE. Bootstrap support values (%, based on 1000 replicates) are shown for each branch. Relative evolutionary rates were calculated as the ratio of root‐to‐tip length to total tree length, reflecting the proportion of evolutionary change each branch contributes to the overall divergence among sequences. The rooting position is based on Cavalier‐Smith et al. (2018).

**Figure 4:**
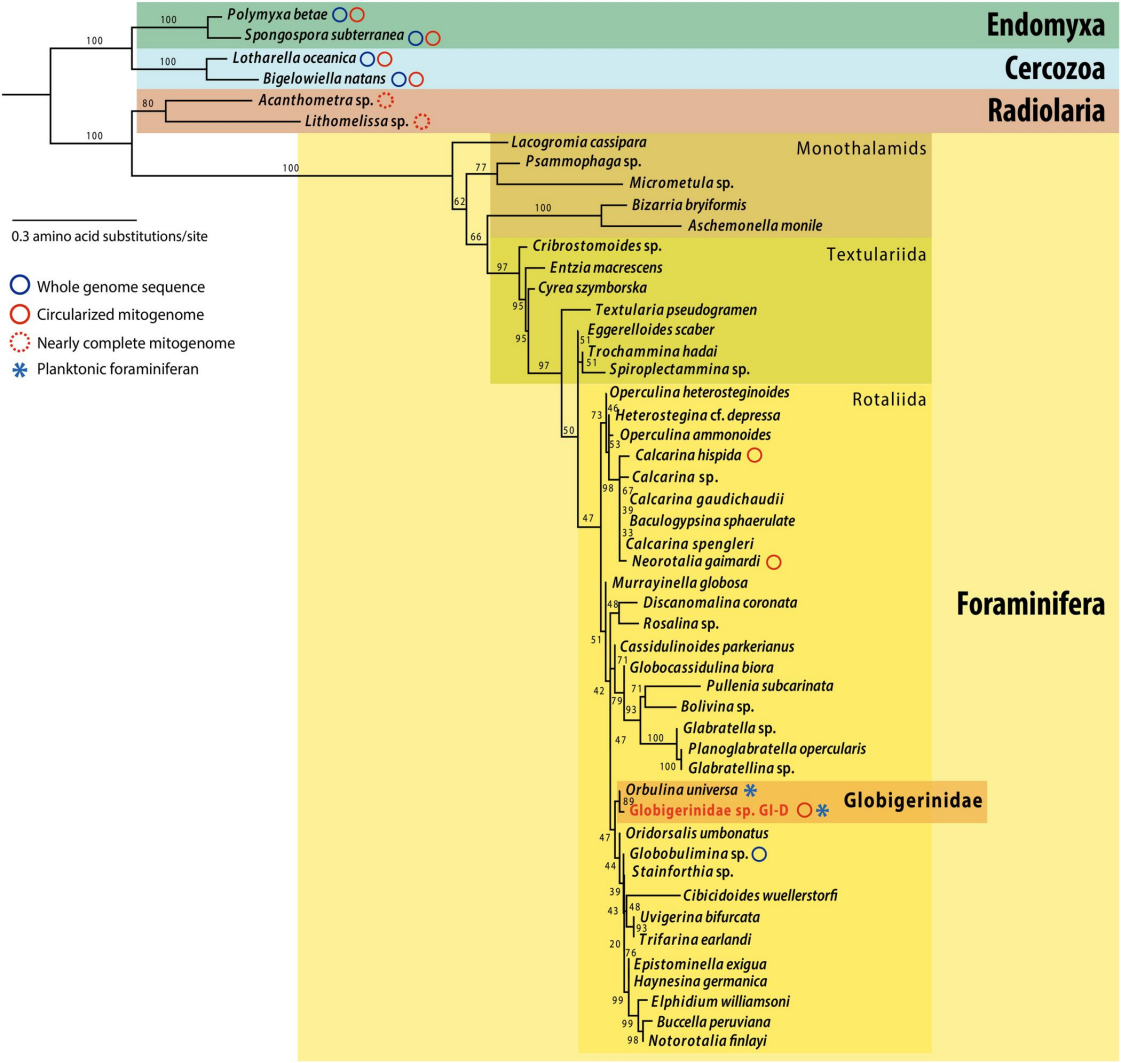
Mitochondrial COX1 phylogeny among Rhizaria. The maximum likelihood tree was reconstructed from COX1 protein sequences of 50 rhizarians (1240 aligned amino acid sites) using IQ‐TREE, with the JTT + F + G4 model. Bootstrap support values (%, based on 1000 replicates) are shown for each branch. The planktonic foraminiferan GI‐D is the most closely related to *Orbulina universa*, which belongs to the family Globigerinidae (order Rotaliida). Blue circles denote species with whole‐genome sequence records, red circles represent the assembly of circularized mitogenomes, and dotted circles indicate the assembly of nearly complete mitogenomes. The planktonic foraminiferans are labeled with asterisks.

A mitogenome tree of Rhizaria was reconstructed from whole‐mitogenome protein sequences (Figure 3), showing the grouping of Globigerinidae sp. GI‐D with the two benthic foraminiferans *C. hispida* and *N. gaimardi*. To expand taxon sampling of foraminiferans beyond those with whole‐mitogenome sequences, another tree was built from the mitochondrial COX1 sequence, which has a good balance of conservation and variation for resolving relationships among foraminiferans (Pentinsaari et al. 2016). The COX1 tree suggests that GI‐D is sister to *Orbulina universa* (Orbigny 1839), which is a known member of the family Globigerinidae (Carpenter et al. 1862) (Figure 4). The comparative genomic (Figure 2) and phylogenetic (Figures 3 and 4) analyses of mitogenomes also suggest that the two benthic species, which both belong to the family Calcarinidae, are closer to each other than to GI‐D.

Branch length analyses of the phylogenomic tree based on the proteins COX1, COX2, NAD1, and NAD7 suggest that the evolutionary rates of globothalamid foraminiferan mitogenomes are faster than those of cercozoans and radiolarians (Figure 3). Further inspection of individual mitochondrial genes reveals that *cox1*, *cox2*, *cox3*, *cob*, *nad1*, *nad3*, *nad4*, *nad4L*, and *nad7* exhibit particularly high evolutionary rates in these foraminiferans, while *nad2* shows comparable rates to those observed in cercozoans and radiolarians (Figure S3).

## 4 Discussion

**Figure 5:**
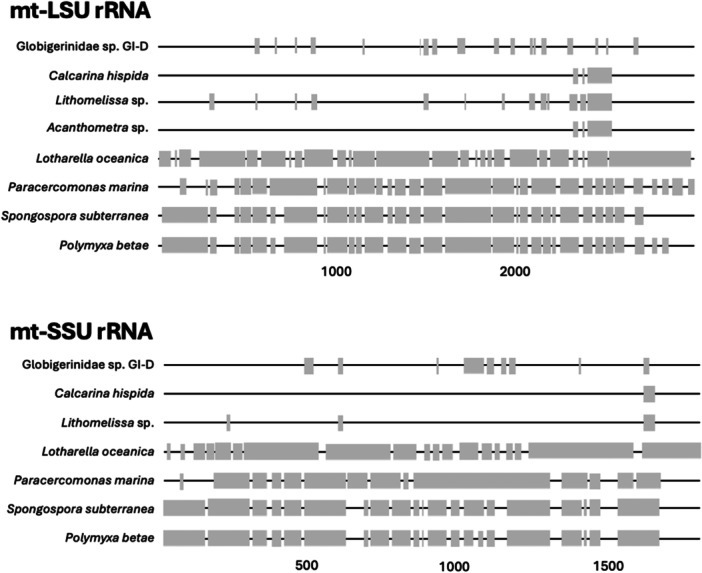
Schematic of mitochondrial (mt) ribosomal RNA (rRNA) alignment. Mitochondrial large‐subunit (LSU) and small‐subunit (SSU) ribosomal RNA genes from representative rhizarians were aligned. Gray blocks represent regions aligned positions, while the black lines indicate unaligned or missing regions. The alignment length (site in bp) is shown along the x‐axis.

**Figure 6:**
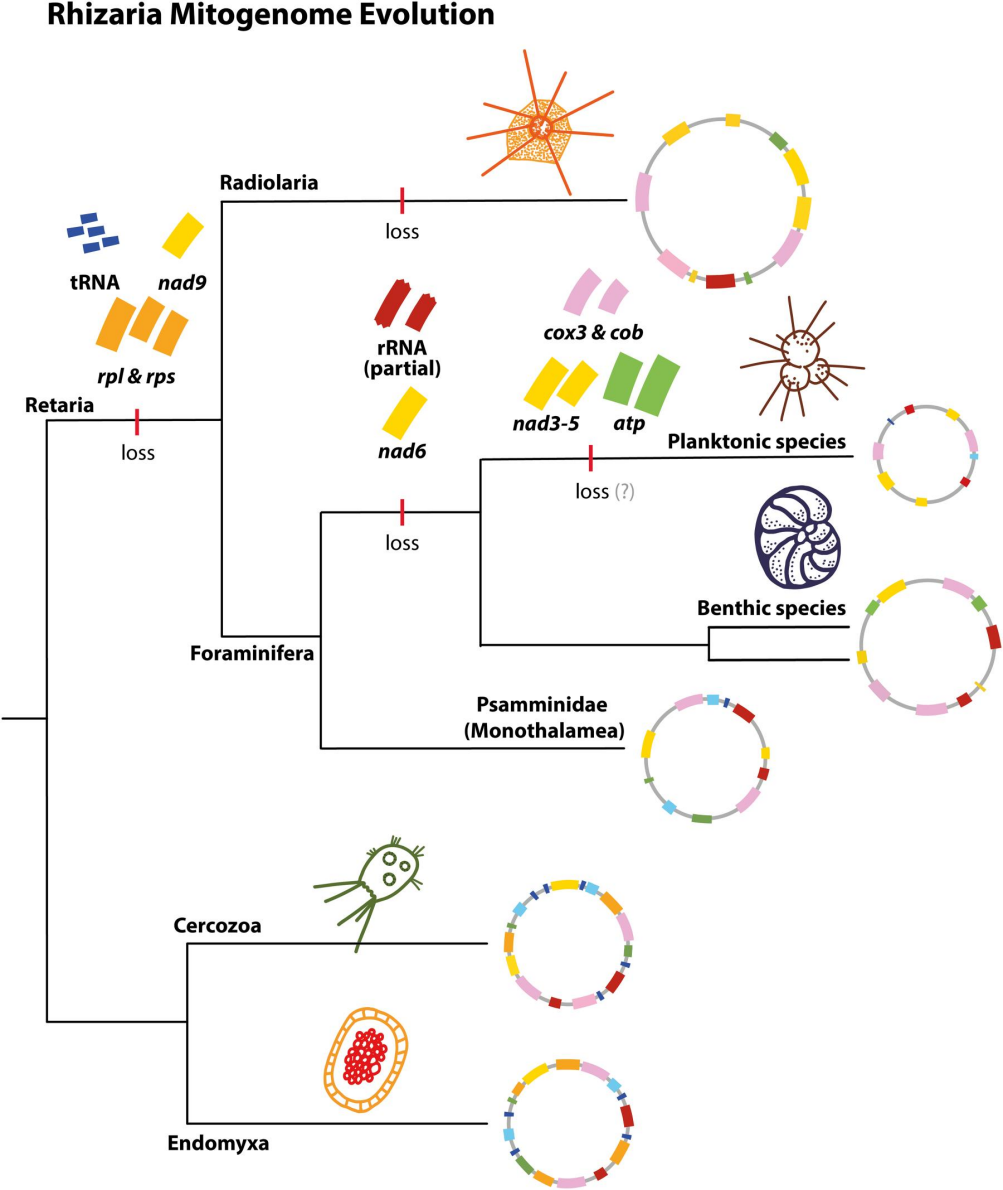
Summary of Rhizaria mitogenome evolution. The mitogenomes of Retaria (Foraminifera and Radiolaria) show a reduction in multiple gene categories, including transfer RNAs (tRNAs), ribosomal proteins, *nad6*, *nad9*, and ribosomal RNAs (rRNAs). In planktonic foraminifera, this reduction is even more pronounced, with the complete loss of *atp* subunit genes.

Through phylogenetic analyses of rhizarian mitogenomes, this study highlights the reductive and accelerated evolution across foraminiferan mitogenomes. Compared with the two previously reported benthic species mitogenomes (Macher et al. 2023), the mitogenome of the planktonic Globigerinidae sp. GI‐D is smaller, with a greater reduction in protein‐coding genes and lower GC content. Such association between accelerated evolutionary rates, genome reduction, and AT richness is well known in prokaryotic genome evolution (Dufresne et al. 2005; McCutcheon and Moran 2012). The mitogenomes of retarians have completely lost key components, including ribosomal proteins, multiple tRNAs, and ETC genes such as *nad6* (except for monothalamid foraminiferans) and *nad9*. In addition, their ribosomal RNA genes are notably fragmented (Figure 5). We note that there is an overall evolutionary trend of gene reduction and accelerated evolution in Rhizaria mitogenomes, from Cercozoa and Endomyxa to Retaria, from Radiolaria to Foramnifera, and from benthic to planktonic foraminiferans (Figure 6). The most extreme case of mitogenome reduction is thus represented by the planktonic foraminiferan GI‐D, which retains only genes encoding a few components of ETC complexes I and IV.

Rapid evolutionary rates have been reported for the nuclear SSU rDNA of planktonic foraminiferans, with gene lengths ranging from 977 bp in *O. universa* to 1178 bp in *Globorotalia menardii* (de Vargas et al. 1997). This accelerated and reductive evolution has been attributed to their shorter reproductive cycles and planktonic lifestyle. In addition, increased molecular evolutionary rates in planktonic microeukaryotes have been linked to fluctuating seawater temperatures, as observed in the genome of marine phytoplankton *Thalassiosira pseudonana* (Schaum et al. 2018). Given that planktonic foraminiferan inhabit a dynamic ecosystem characterized by variable temperature, salinity and nutrient conditions, their higher sensitivity to environmental fluctuations, including ocean currents and weather patterns (Wei and Kennett 1986; Malmgren and Berggren 1987), may contribute to an elevated substitution rate and lineage‐specific evolutionary patterns at the molecular level, which is a topic that merits further investigation.

Similar to Foraminifera mitogenomes, pervasive gene loss is observed in another SAR lineage, Myzozoa (Alveolata), which includes apicoplexans, dinoflagellates, and chrompodellids. Myzozoans possess highly reduced mitogenomes, with only ETC complex III and IV genes and fragmented ribosomal RNA genes (Waller and Jackson 2009; Nash et al. 2008; Feagin et al. 2012). This pattern of rRNA fragmentation is also found in distantly related lineages, such as the green alga *Polytomella magna* (Chlorophyta), where a reduced mitochondrial rRNA consists of 13 fragments (Tobiasson et al. 2022). A cryo‐EM study found that the rRNA in the *Toxoplasma* (apicomplexan) ribosome has over 50 molecules, with repetitive use of the same sequences, addition of poly‐A tails, and interaction with non‐ribosomal proteins repurposed as components of the mitochondrial ribosome (Shikha et al. 2025). The widespread convergence of mitochondrial rRNA gene fragmentation and reduction across distant lineages suggests this is a repeated pattern in eukaryote evolution. While myzozoan rRNA fragmentation and massive gene loss have been attributed to an ancestral linearized alveolate mitogenome (Flegontov et al. 2015), this may not apply to globothalamid Foraminifera, given that all currently sequenced Rhizarian mitogenomes are circular. This highlights Foraminifera as an important example for mitogenome evolution. It would be intriguing to investigate if certain cellular or life cycle features are associated with mitogenome reduction and rRNA fragmentation.

Compared with benthic foraminiferans, the GI‐D mitogenome has a higher AT content, a higher proportion of repetitive sequences (Table 2), and an additional GIY‐YIG endonuclease gene (Figure 1). Given that repetitive sequences can act as recombination hotspots (Aras et al. 2003), a higher density of repetitive elements suggests an enhanced potential for recombination‐driven genome rearrangement in planktonic foraminiferan mitogenomes. GIY‐YIG endonuclease family genes are prevalent in bacteria and have also been identified in the mitogenomes of various eukaryotes, including fungi (Saguez 2000; Megarioti and Kouvelis 2020) and protists (Craig et al. 2021). Given that endonuclease genes have been implicated in DNA cleavage, repair, and recombination (Aravind 1999; Mak et al. 2010), their presence can also contribute to mitogenome structural changes. This is consistent with the observation that GI‐D and the previously reported benthic foraminiferan mitogenomes (Macher et al. 2023) have little conservation in genome organization. Such mitogenome features and plasticity may be associated with adaptation to environmental stress in marine planktonic environments that are characterized by fluctuations in temperature, oxygen, and other parameters.

The distinct mitogenome gene repertoire might shed light on the physiology of planktonic foraminiferans. The lack of several genes typically encoded by mitogenomes of aerobic eukaryotes (*cox3*, *cob*, *nad3*, *nad4*, *nad4L*, *nad5*, *nad6*, *atp6*, *atp8*, and *atp9*) (Butenko et al. 2024) suggests it might not have typical aerobic energy metabolism. A recent study indicates that anaerobic respiration can occur in more anoxic microenvironments inside planktonic foraminiferan particles, even at depths above the marine oxygen minimum zone (Doherty et al. 2025). It has also been shown that anaerobic metabolism can provide all the energy needed for necessary cellular functions in benthic foraminiferans *Bolivina* and *Stainforthia* (Orsi et al. 2020), which are among the benthic foraminiferan relatives of Globigerinidae. Given that planktonic foraminiferans are derived from benthic ancestors, anaerobic metabolism can still play a physiological role for planktonic foraminiferans. We also note that several lineages of photosynthetic eukaryotes are known for the complete loss of some core respiration genes, including *Euglena* (several complex I genes), chromerids and dinoflagellates (complex I genes) (Butenko et al. 2024), and a few Stylonematophyceae red algae (*nad3*, *nad4L*, *nad6*, and *atp8*) (Lee et al. 2023).

For GI‐D, the absence of those genes indicates either that they have been completely lost from GI‐D or that some of those typically mitogenome‐encoded genes have been transferred to and are now encoded by the nuclear genome, which is rare among eukaryotes (Butenko et al. 2024). Because the nuclear genome of GI‐D was difficult to sequence and assemble using the current SAG approach, we do not have direct evidence supporting either of these two scenarios. Here, if we assume they are completely absent from both the mitogenome and the nuclear genome of GI‐D, we could imagine that planktonic foraminiferans have a rather unique mitochondrial metabolism. Due to the lack of ATP synthase subunits, GI‐D would rely on substrate‐level phosphorylation for ATP production, similar to the alveolate *Nyctotherus* and stramenopile *Blastocystis* (de Graaf et al. 2011). Moreover, the absence of cytochrome b (complex III) would mean that there is a kind of disconnected ETC, which has been observed in the photosynthetic alveolate *Chromera* (Oborník and Lukeš 2015). Given the lack of ATP synthase, the redox energy of this bipartite ETC would not be channeled into a proton gradient, but linked to substrate‐level phosphorylation. Finally, we note that for complexes I and IV, GI‐D has a much shorter NADH dehydrogenase subunit 1 and cytochrome c oxidase subunit 2 than the benthic foraminiferans and other rhizarians (Figure 2), which further underscores the uniqueness of GI‐D mitochondria, which may be characterized by a lack of ATP synthase, a bipartite ETC, and modified ETC complexes. Although we did not find other foraminiferan mitogenome contigs containing additional ETC genes, an alternative explanation for the loss of ETC genes in GI‐D is that another mitogenome sequence that contains these genes but remains undetected, given that these mitochondrial ETC proteins are typically encoded by the mitogenome and rarely lost. Such a bipartite or multipartite mitogenome, if present, would also distinguish GI‐D from other rhizarians. In any case, further research is needed to improve our knowledge of the genomics, mitochondrial biology, metabolism, and respiratory chain biochemistry of planktonic foraminiferans.

Rhizarians exhibit a diverse range of mitochondria or mitochondrion‐related organelles (MROs). Mitosomes without mitogenome DNA are found in the animal‐parasitic endomyxans *Mikrocytos* and *Paramikrocytos* (Burki et al. 2013; Onuț‐Brännström et al. 2023; Žárský et al. 2023). The anaerobic, free‐living cercozoan, *Brevimastigomonas motovehiculus*, possesses hydrogenosome‐like organelles with a reduced mitogenome and ETC (without complexes III and IV), which can be early adaptations to low oxygen (Gawryluk et al. 2016). The endomyxans SSF and PG, which are recently discovered anaerobic, free‐living rhizarians, have also been presumed to have more anaerobic MROs with a certain degree of mitochondrial reduction (Eglit et al. 2024). In this study, we further suggest that planktonic foraminiferans likely represent another example of mitogenome reductive evolution in Rhizaria, with a small gene repertoire, accelerated evolutionary rates, rRNA fragmentation, low GC content, high repeat density, and low conservation in genome organization. The physiological significance of the reduction and variation of mitogenomes in foraminiferans and the implications of these genomic features for the biology of foraminiferans merit further studies.

*Notes:* The mitogenome sequences associated with two xenophyophore foraminiferans, *S. delicata* and *P.* aff. *limbata* (Psamminidae, Monothalamea) (Gastineau et al. 2025), were added to the analyses during the editorial revision of the paper.

## Author Contributions

**Hsin‐Tung Lai:** writing – review and editing, formal analysis, visualization, writing – original draft, conceptualization, investigation. **Ming‐Wei Lai:** investigation, validation, visualization, writing – review and editing, formal analysis. **Tzu‐Haw Wang:** software, methodology, validation. **Haojia Ren:** methodology, funding acquisition, resources. **Chuan Ku:** writing – review and editing, writing – original draft, methodology, conceptualization, funding acquisition, project administration, visualization, validation, investigation, supervision.

## Conflicts of Interest

None declared.

## Supporting information


**Figure S1:** Mitochondrial COX1 protein phylogeny of Foraminifera, including three particles from Green Island (GI) seawater.
**Figure S2:** The frequency of repetitive sequences in the mitogenomes of globothalmid foraminiferans.
**Figure S3:** Individual mitochondrial gene trees of Rhizaria.
**Table S1:** Complete or nearly complete rhizarian mitogenome sequences analyzed in this study.
**Table S2:** Taxonomic distribution of contigs (%) in each planktonic foraminiferan (Globigerinidae) particle SAG.

## Data Availability

The mitogenome sequences of Globigerinidae sp. GI‐D and its associated *Chrysochromulina* sp. were deposited in the NCBI with the GenBank accession numbers PV558267 and PV535690, respectively. Their associated BioProject accession is PRJNA1251237.

## References

[mbo370368-bib-0001] Adl, S. M. , D. Bass , C. E. Lane , et al. 2019. “Revisions to the Classification, Nomenclature, and Diversity of Eukaryotes.” Journal of Eukaryotic Microbiology 66, no. 1: 4–119. 10.1111/jeu.12691. 30257078 PMC6492006

[mbo370368-bib-0002] Aras, R. A. , J. Kang , A. I. Tschumi , Y. Harasaki , and M. J. Blaser . 2003. “Extensive Repetitive DNA Facilitates Prokaryotic Genome Plasticity.” Proceedings of the National Academy of Sciences 100, no. 23: 13579–13584. 10.1073/pnas.1735481100. PMC263856 14593200

[mbo370368-bib-0003] Aravind, L. 1999. “Conserved Domains in DNA Repair Proteins and Evolution of Repair Systems.” Nucleic Acids Research 27, no. 5: 1223–1242. 10.1093/nar/27.5.1223. 9973609 PMC148307

[mbo370368-bib-0004] Archibald, J. M. 2015. “Endosymbiosis and Eukaryotic Cell Evolution.” Current Biology 25, no. 19: R911–R921. 10.1016/j.cub.2015.07.055. 26439354

[mbo370368-bib-0005] Avvaru, A. K. , D. T. Sowpati , and R. K. Mishra . 2018. “PERF: An Exhaustive Algorithm for Ultra‐Fast and Efficient Identification of Microsatellites From Large DNA Sequences.” Bioinformatics 34, no. 6: 943–948. 10.1093/bioinformatics/btx721. 29121165

[mbo370368-bib-0006] Bernt, M. , A. Donath , F. Jühling , et al. 2013. “MITOS: Improved De Novo Metazoan Mitochondrial Genome Annotation.” Molecular Phylogenetics and Evolution 69, no. 2: 313–319. 10.1016/j.ympev.2012.08.023. 22982435

[mbo370368-bib-0007] Burger, G. , M. W. Gray , L. Forget , and B. F. Lang . 2013. “Strikingly Bacteria‐Like and Gene‐Rich Mitochondrial Genomes Throughout Jakobid Protists.” Genome Biology and Evolution 5, no. 2: 418–438. 10.1093/gbe/evt008. 23335123 PMC3590771

[mbo370368-bib-0008] Burki, F. , N. Corradi , R. Sierra , et al. 2013. “Phylogenomics of the Intracellular Parasite *Mikrocytos mackini* Reveals Evidence for a Mitosome in Rhizaria.” Current Biology 23, no. 16: 1541–1547. 23891116 10.1016/j.cub.2013.06.033

[mbo370368-bib-0009] Butenko, A. , J. Lukeš , D. Speijer , and J. G. Wideman . 2024. “Mitochondrial Genomes Revisited: Why Do Different Lineages Retain Different Genes?” BMC Biology 22, no. 1: 15. 10.1186/s12915-024-01824-1. 38273274 PMC10809612

[mbo370368-bib-0010] Cai‐Li, R.‐Y. , H. Ren , W.‐N. Fang , et al. 2025. “Symbiont Regulation of Nitrogen Metabolism and Excretion in Tropical Planktonic Foraminifera.” Geochimica et Cosmochimica Acta 396: S0016703725001255. 10.1016/j.gca.2025.03.009.

[mbo370368-bib-0011] Camacho, C. , G. Coulouris , V. Avagyan , et al. 2009. “BLAST+: Architecture and Applications.” BMC Bioinformatics 10, no. 1: 421. 10.1186/1471-2105-10-421. 20003500 PMC2803857

[mbo370368-bib-0012] Carpenter, W. B. , W. R. Parker , T. R. Jones , and R. Hardwicke . 1862. “Introduction to the Study of the Foraminifera.” Journal of Cell Science S2–2, no. 8: 297–301.

[mbo370368-bib-0013] Cavalier‐Smith, T. , E. E. Chao , and R. Lewis . 2018. “Multigene Phylogeny and Cell Evolution of Chromist Infrakingdom Rhizaria: Contrasting Cell Organisation of Sister Phyla Cercozoa and Retaria.” Protoplasma 255, no. 5: 1517–1574. 10.1007/s00709-018-1241-1. 29666938 PMC6133090

[mbo370368-bib-0014] Chen, S. , Y. Zhou , Y. Chen , and J. Gu . 2018. “fastp: An Ultra‐Fast All‐in‐One FASTQ Preprocessor.” Bioinformatics 34, no. 17: i884–i890. 10.1093/bioinformatics/bty560. 30423086 PMC6129281

[mbo370368-bib-0015] Chinnery, P. F. , and G. Hudson . 2013. “Mitochondrial Genetics.” British Medical Bulletin 106, no. 1: 135–159. 10.1093/bmb/ldt017. 23704099 PMC3675899

[mbo370368-bib-0016] Ciobanu, D. , A. Clum , S. Ahrendt , et al. 2021. “A Single‐Cell Genomics Pipeline for Environmental Microbial Eukaryotes.” iScience 24, no. 4: 102290. 10.1016/j.isci.2021.102290. 33870123 PMC8042348

[mbo370368-bib-0017] Craig, R. J. , I. A. Yushenova , F. Rodriguez , and I. R. Arkhipova . 2021. “An Ancient Clade of *Penelope*‐Like Retroelements With Permuted Domains Is Present in the Green Lineage and Protists, and Dominates Many Invertebrate Genomes.” Molecular Biology and Evolution 38, no. 11: 5005–5020. 10.1093/molbev/msab225. 34320655 PMC8557442

[mbo370368-bib-0018] de Graaf, R. M. , G. Ricard , and T. A. van Alen , et al. 2011. “The Organellar Genome and Metabolic Potential of the Hydrogen‐Producing Mitochondrion of *Nyctotherus ovalis* .” Molecular Biology and Evolution 28, no. 8: 2379–2391. 10.1093/molbev/msr059. 21378103 PMC3144386

[mbo370368-bib-0019] de Vargas, C. , L. Zaninetti , H. Hilbrecht , and J. Pawlowski . 1997. “Phylogeny and Rates of Molecular Evolution of Planktonic Foraminifera: SSU rDNA Sequences Compared to the Fossil Record.” Journal of Molecular Evolution 45, no. 3: 285–294. 10.1007/PL00006232. 9302323

[mbo370368-bib-0020] Dierckxsens, N. , P. Mardulyn , and G. Smits . 2016. “NOVOPlasty: *De Novo* Assembly of Organelle Genomes From Whole Genome Data.” Nucleic Acids Research 45: gkw955. 10.1093/nar/gkw955. PMC5389512 28204566

[mbo370368-bib-0021] Dobáková, E. , P. Flegontov , T. Skalický , and J. Lukeš . 2015. “Unexpectedly Streamlined Mitochondrial Genome of the Euglenozoan *Euglena gracilis* .” Genome Biology and Evolution 7, no. 12: 3358–3367. 10.1093/gbe/evv229. 26590215 PMC4700960

[mbo370368-bib-0022] Doherty, S. C. , C. V. Davis , and J. S. Fehrenbacher . 2025. “Planktic Foraminifera Record the Succession of Anaerobic Metabolisms in Particle Microenvironments Across a Pelagic Oxygen Gradient.” Geochimica et Cosmochimica Acta 395: 267–276.

[mbo370368-bib-0023] Dufresne, A. , L. Garczarek , and F. Partensky . 2005. “Accelerated Evolution Associated With Genome Reduction in a Free‐Living Prokaryote.” Genome Biology 6, no. 2: R14. 10.1186/gb-2005-6-2-r14. 15693943 PMC551534

[mbo370368-bib-0024] Eglit, Y. , M. Lawton , A. G. B. Simpson , and R. M. R. Gawryluk. 2024. “Two Newly Cultivated Eukaryotrophic Flagellates Represent Distinct Anaerobic Lineages Within Rhizaria.” bioRxiv. 10.1101/2024.09.01.610664.

[mbo370368-bib-0025] Emms, D. M. , and S. Kelly . 2019. “OrthoFinder: Phylogenetic Orthology Inference for Comparative Genomics.” Genome Biology 20, no. 1: 238. 10.1186/s13059-019-1832-y. 31727128 PMC6857279

[mbo370368-bib-0026] Fang, W.‐N. , O. Branson , E.‐W. Yang , et al. 2025. “Direct Pathway of Incorporating Dietary Nitrogen in Shell‐Bound Matrix of the Planktic Foraminifera *Trilobatus sacculifer* .” Earth and Planetary Science Letters 654: 119231. 10.1016/j.epsl.2025.119231.

[mbo370368-bib-0027] Feagin, J. E. , M. I. Harrell , J. C. Lee , et al. 2012. “The Fragmented Mitochondrial Ribosomal RNAs of *Plasmodium falciparum* .” PLoS ONE 7, no. 6: e38320. 10.1371/journal.pone.0038320. 22761677 PMC3382252

[mbo370368-bib-0028] Flegontov, P. , J. Michálek , J. Janouškovec , et al. 2015. “Divergent Mitochondrial Respiratory Chains in Phototrophic Relatives of Apicomplexan Parasites.” Molecular Biology and Evolution 32, no. 5: 1115–1131. 10.1093/molbev/msv021. 25660376

[mbo370368-bib-0029] Gastineau, R. , K. Mianowicz , P. Dąbek , et al. 2025. “Xenophyophore‐Associated Mitogenomes: Genomic Investigations of Two Specimens From the Clarion‐Clipperton Zone.” Frontiers in Marine Science 12: 1582660. 10.3389/fmars.2025.1582660.

[mbo370368-bib-0030] Gawryluk, R. M. R. , R. Kamikawa , C. W. Stairs , J. D. Silberman , M. W. Brown , and A. J. Roger . 2016. “The Earliest Stages of Mitochondrial Adaptation to Low Oxygen Revealed in a Novel Rhizarian.” Current Biology 26, no. 20: 2729–2738. 10.1016/j.cub.2016.08.025. 27666965

[mbo370368-bib-0031] Glöckner, G. , N. Hülsmann , M. Schleicher , et al. 2014. “The Genome of the Foraminiferan *Reticulomyxa filosa* .” Current Biology 24, no. 1: 11–18. 10.1016/j.cub.2013.11.027. 24332546

[mbo370368-bib-0032] Gray, M. W. 2014. “The Pre‐Endosymbiont Hypothesis: A New Perspective on the Origin and Evolution of Mitochondria.” Cold Spring Harbor Perspectives in Biology 6, no. 3: a016097. 10.1101/cshperspect.a016097. 24591518 PMC3949359

[mbo370368-bib-0033] Greiner, S. , P. Lehwark , and R. Bock . 2019. “OrganellarGenomeDRAW (OGDRAW) Version 1.3.1: Expanded Toolkit for the Graphical Visualization of Organellar Genomes.” Nucleic Acids Research 47, no. W1: W59–W64. 10.1093/nar/gkz238. 30949694 PMC6602502

[mbo370368-bib-0034] Gurevich, A. , V. Saveliev , N. Vyahhi , and G. Tesler . 2013. “QUAST: Quality Assessment Tool for Genome Assemblies.” Bioinformatics 29, no. 8: 1072–1075. 10.1093/bioinformatics/btt086. 23422339 PMC3624806

[mbo370368-bib-0035] Habura, A. , Y. Hou , A. A. Reilly , and S. S. Bowser . 2011. “High‐Throughput Sequencing of *Astrammina rara*: Sampling the Giant Genome of a Giant Foraminiferan Protist.” BMC Genomics 12, no. 1: 169. 10.1186/1471-2164-12-169. 21453490 PMC3079666

[mbo370368-bib-0036] Janouškovec, J. , D. V. Tikhonenkov , F. Burki , et al. 2017. “A New Lineage of Eukaryotes Illuminates Early Mitochondrial Genome Reduction.” Current Biology 27, no. 23: 3717–3724.e5. 10.1016/j.cub.2017.10.051. 29174886

[mbo370368-bib-0037] Kalyaanamoorthy, S. , B. Q. Minh , T. K. F. Wong , A. Von Haeseler , and L. S. Jermiin . 2017. “ModelFinder: Fast Model Selection for Accurate Phylogenetic Estimates.” Nature Methods 14, no. 6: 587–589. 10.1038/nmeth.4285. 28481363 PMC5453245

[mbo370368-bib-0038] Karlicki, M. , S. Antonowicz , and A. Karnkowska . 2022. “Tiara: Deep Learning‐Based Classification System for Eukaryotic Sequences.” Bioinformatics 38, no. 2: 344–350. 10.1093/bioinformatics/btab672. 34570171 PMC8722755

[mbo370368-bib-0039] Katoh, K. , and D. M. Standley . 2013. “MAFFT Multiple Sequence Alignment Software Version 7: Improvements in Performance and Usability.” Molecular Biology and Evolution 30, no. 4: 772–780. 10.1093/molbev/mst010. 23329690 PMC3603318

[mbo370368-bib-0040] Keeling, P. J. 2019. “Combining Morphology, Behaviour and Genomics to Understand the Evolution and Ecology of Microbial Eukaryotes.” Philosophical Transactions of the Royal Society, B: Biological Sciences 374, no. 1786: 20190085. 10.1098/rstb.2019.0085. PMC6792444 31587641

[mbo370368-bib-0041] Ku, C. , S. Nelson‐Sathi , M. Roettger , et al. 2015. “Endosymbiotic Origin and Differential Loss of Eukaryotic Genes.” Nature 524, no. 7566: 427–432. 26287458 10.1038/nature14963

[mbo370368-bib-0042] Ku, C. , and A. Sebé‐Pedrós . 2019. “Using Single‐Cell Transcriptomics to Understand Functional States and Interactions in Microbial Eukaryotes.” Philosophical Transactions of the Royal Society, B: Biological Sciences 374, no. 1786: 20190098. 10.1098/rstb.2019.0098. PMC6792447 31587645

[mbo370368-bib-0043] Kucera, M. 2007. “Chapter Six Planktonic Foraminifera as Tracers of Past Oceanic Environments.” Developments in Marine Geology 1: 213–262. 10.1016/S1572-5480(07)01011-1.

[mbo370368-bib-0044] Lang, B. F. , N. Beck , S. Prince , M. Sarrasin , P. Rioux , and G. Burger . 2023. “Mitochondrial Genome Annotation With MFannot: A Critical Analysis of Gene Identification and Gene Model Prediction.” Frontiers in Plant Science 14: 1222186. 10.3389/fpls.2023.1222186. 37469769 PMC10352661

[mbo370368-bib-0045] Langmead, B. , and S. L. Salzberg . 2012. “Fast Gapped‐Read Alignment With Bowtie 2.” Nature Methods 9, no. 4: 357–359. 10.1038/nmeth.1923. 22388286 PMC3322381

[mbo370368-bib-0046] Lee, Y. , C. H. Cho , C. Noh , et al. 2023. “Origin of Minicircular Mitochondrial Genomes in Red Algae.” Nature Communications 14: 3363. 10.1038/s41467-023-39084-2 PMC10250338 37291154

[mbo370368-bib-0047] Li, H. 2018. “Minimap2: Pairwise Alignment for Nucleotide Sequences.” Bioinformatics 34, no. 18: 3094–3100. 10.1093/bioinformatics/bty191. 29750242 PMC6137996

[mbo370368-bib-0048] Macher, J.‐N. , D. M. Bloska , M. Holzmann , E. B. Girard , J. Pawlowski , and W. Renema . 2022. “Mitochondrial Cytochrome c Oxidase Subunit I (COI) Metabarcoding of Foraminifera Communities Using Taxon‐Specific Primers.” PeerJ 10: e13952. 10.7717/peerj.13952. 36093332 PMC9454970

[mbo370368-bib-0049] Macher, J.‐N. , N. L. Coots , Y.‐P. Poh , et al. 2023. “Single‐Cell Genomics Reveals the Divergent Mitochondrial Genomes of Retaria (Foraminifera and Radiolaria).” mBio 14, no. 2: e00302‐23. 10.1128/mbio.00302-23. 36939357 PMC10127745

[mbo370368-bib-0050] Mak, A. N.‐S. , A. R. Lambert , and B. L. Stoddard . 2010. “Folding, DNA Recognition, and Function of GIY‐YIG Endonucleases: Crystal Structures of R.Eco29kI.” Structure 18, no. 10: 1321–1331. 10.1016/j.str.2010.07.006. 20800503 PMC2955809

[mbo370368-bib-0051] Malmgren, B. A. , and W. A. Berggren . 1987. “Evolutionary Changes in Some Late Neogene Planktonic Foraminiferal Lineages and Their Relationships to Paleoceanographic Changes.” Paleoceanography 2, no. 5: 445–456. 10.1029/PA002i005p00445.

[mbo370368-bib-0052] McCutcheon, J. P. , and N. A. Moran . 2012. “Extreme Genome Reduction in Symbiotic Bacteria.” Nature Reviews Microbiology 10, no. 1: 13–26. 10.1038/nrmicro2670. 22064560

[mbo370368-bib-0053] Megarioti, A. H. , and V. N. Kouvelis . 2020. “The Coevolution of Fungal Mitochondrial Introns and Their Homing Endonucleases (GIY‐YIG and LAGLIDADG).” Genome Biology and Evolution 12, no. 8: 1337–1354. 10.1093/gbe/evaa126. 32585032 PMC7487136

[mbo370368-bib-0054] Mohamed Yusoff, A. A. , S. Z. N. Mohd Khair , and S. M. Abd Radzak . 2025. “Mitochondrial DNA Copy Number Alterations: Key Players in the Complexity of Glioblastoma (Review).” Molecular Medicine Reports 31, no. 3: 78. 10.3892/mmr.2025.13443. 39886971 PMC11795256

[mbo370368-bib-0055] Morard, R. , K. F. Darling , A. K. M. Weiner , et al. 2024. “The Global Genetic Diversity of Planktonic Foraminifera Reveals the Structure of Cryptic Speciation in Plankton.” Biological Reviews 99, no. 4: 1218–1241. 10.1111/brv.13065. 38351434

[mbo370368-bib-0056] Müller, M. , M. Mentel , J. J. Van Hellemond , et al. 2012. “Biochemistry and Evolution of Anaerobic Energy Metabolism in Eukaryotes.” Microbiology and Molecular Biology Reviews 76, no. 2: 444–495. 10.1128/MMBR.05024-11. 22688819 PMC3372258

[mbo370368-bib-0057] Nash, E. A. , R. E. R. Nisbet , A. C. Barbrook , and C. J. Howe . 2008. “Dinoflagellates: A Mitochondrial Genome All at Sea.” Trends in Genetics 24, no. 7: 328–335. 10.1016/j.tig.2008.04.001. 18514360

[mbo370368-bib-0058] Nguyen, L.‐T. , H. A. Schmidt , A. Von Haeseler , and B. Q. Minh . 2015. “IQ‐TREE: A Fast and Effective Stochastic Algorithm for Estimating Maximum‐Likelihood Phylogenies.” Molecular Biology and Evolution 32, no. 1: 268–274. 10.1093/molbev/msu300. 25371430 PMC4271533

[mbo370368-bib-0059] Oborník, M. , and J. Lukeš . 2015. “The Organellar Genomes of *Chromera* and *Vitrella*, the Phototrophic Relatives of Apicomplexan Parasites.” Annual Review of Microbiology 69: 129–144. 10.1146/annurev-micro-091014-104449. 26092225

[mbo370368-bib-0060] Onuț‐Brännström, I. , C. W. Stairs , K. I. A. Campos , et al. 2023. “A Mitosome With Distinct Metabolism in the Uncultured Protist Parasite *Paramikrocytos canceri* (Rhizaria, Ascetosporea).” Genome Biology and Evolution 15, no. 3: evad022. 10.1093/gbe/evad022. 36790104 PMC9998036

[mbo370368-bib-0061] Orbigny, A. D. d’ . 1839. Foraminifères [of Cuba], Vol. 8. Bertrand.

[mbo370368-bib-0062] Orsi, W. D. , R. Morard , A. Vuillemin , et al. 2020. “Anaerobic Metabolism of Foraminifera Thriving Below the Seafloor.” ISME Journal 14: 2580–2594. 32641728 10.1038/s41396-020-0708-1 PMC7490399

[mbo370368-bib-0063] Pawlowski, J. , M. Holzmann , and J. Tyszka . 2013. “New Supraordinal Classification of Foraminifera: Molecules Meet Morphology.” Marine Micropaleontology 100: 1–10. 10.1016/j.marmicro.2013.04.002.

[mbo370368-bib-0064] Pentinsaari, M. , H. Salmela , M. Mutanen , and T. Roslin . 2016. “Molecular Evolution of a Widely‐Adopted Taxonomic Marker (COI) Across the Animal Tree of Life.” Scientific Reports 6, no. 1: 35275. 10.1038/srep35275. 27734964 PMC5062346

[mbo370368-bib-0065] Prjibelski, A. , D. Antipov , D. Meleshko , A. Lapidus , and A. Korobeynikov . 2020. “Using SPAdes De Novo Assembler.” Current Protocols in Bioinformatics 70, no. 1: e102. 10.1002/cpbi.102. 32559359

[mbo370368-bib-0066] Rambaut, A. 2012. FigTree v1. 4. Molecular Evolution, Phylogenetics Andepidemiology. University of Edinburgh, Institute of Evolutionary Biology.

[mbo370368-bib-0067] Saguez, C. 2000. “Intronic GIY‐YIG Endonuclease Gene in the Mitochondrial Genome of *Podospora curvicolla*: Evidence for Mobility.” Nucleic Acids Research 28, no. 6: 1299–1306. 10.1093/nar/28.6.1299. 10684923 PMC111034

[mbo370368-bib-0068] Schaum, C.‐E. , A. Buckling , N. Smirnoff , D. J. Studholme , and G. Yvon‐Durocher . 2018. “Environmental Fluctuations Accelerate Molecular Evolution of Thermal Tolerance in a Marine Diatom.” Nature Communications 9, no. 1: 1719. 10.1038/s41467-018-03906-5. PMC5928086 29712900

[mbo370368-bib-0069] Schön, M. E. , V. V. Zlatogursky , R. P. Singh , et al. 2021. “Single Cell Genomics Reveals Plastid‐Lacking Picozoa Are Close Relatives of Red Algae.” Nature Communications 12, no. 1: 6651. 10.1038/s41467-021-26918-0. PMC8599508 34789758

[mbo370368-bib-0070] Shikha, S. , V. Tobiasson , M. Ferreira Silva , et al. 2025. “Numerous rRNA Molecules Form the Apicomplexan Mitoribosome via Repurposed Protein and RNA Elements.” Nature Communications 16, no. 1: 817. 10.1038/s41467-025-56057-9. PMC11742926 39827269

[mbo370368-bib-0071] Smith, D. R. , and P. J. Keeling . 2015. “Mitochondrial and Plastid Genome Architecture: Reoccurring Themes, but Significant Differences at the Extremes.” Proceedings of the National Academy of Sciences 112, no. 33: 10177–10184. 10.1073/pnas.1422049112. PMC4547224 25814499

[mbo370368-bib-0072] Tanifuji, G. , J. M. Archibald , and T. Hashimoto . 2016. “Comparative Genomics of Mitochondria in Chlorarachniophyte Algae: Endosymbiotic Gene Transfer and Organellar Genome Dynamics.” Scientific Reports 6, no. 1: 21016. 10.1038/srep21016. 26888293 PMC4757882

[mbo370368-bib-0073] Thorvaldsdottir, H. , J. T. Robinson , and J. P. Mesirov . 2013. “Integrative Genomics Viewer (IGV): High‐Performance Genomics Data Visualization and Exploration.” Briefings in Bioinformatics 14, no. 2: 178–192. 10.1093/bib/bbs017. 22517427 PMC3603213

[mbo370368-bib-0074] Timmis, J. N. , M. A. Ayliffe , C. Y. Huang , and W. Martin . 2004. “Endosymbiotic Gene Transfer: Organelle Genomes Forge Eukaryotic Chromosomes.” Nature Reviews Genetics 5, no. 2: 123–135. 10.1038/nrg1271. 14735123

[mbo370368-bib-0075] Tobiasson, V. , I. Berzina , and A. Amunts . 2022. “Structure of a Mitochondrial Ribosome With Fragmented rRNA in Complex With Membrane‐Targeting Elements.” Nature Communications 13, no. 1: 6132. 10.1038/s41467-022-33582-5. PMC9576764 36253367

[mbo370368-bib-0076] Waller, R. F. , and C. J. Jackson . 2009. “Dinoflagellate Mitochondrial Genomes: Stretching the Rules of Molecular Biology.” BioEssays 31, no. 2: 237–245. 10.1002/bies.200800164. 19204978

[mbo370368-bib-0077] Wei, K. Y. , and J. P. Kennett . 1986. “Taxonomic Evolution of Neogene Planktonic Foraminifera and Paleoceanographic Relations.” Paleoceanography 1, no. 1: 67–84. 10.1029/PA001i001p00067.

[mbo370368-bib-0078] Wick, R. R. , L. M. Judd , and K. E. Holt . 2018. “Deepbinner: Demultiplexing Barcoded Oxford Nanopore Reads With Deep Convolutional Neural Networks.” PLoS Computational Biology 14, no. 11: e1006583. 10.1371/journal.pcbi.1006583. 30458005 PMC6245502

[mbo370368-bib-0079] Wideman, J. G. , A. Monier , R. Rodríguez‐Martínez , et al. 2019. “Unexpected Mitochondrial Genome Diversity Revealed by Targeted Single‐Cell Genomics of Heterotrophic Flagellated Protists.” Nature Microbiology 5, no. 1: 154–165. 10.1038/s41564-019-0605-4. 31768028

[mbo370368-bib-0080] Woehle, C. , A.‐S. Roy , N. Glock , et al. 2018. “A Novel Eukaryotic Denitrification Pathway in Foraminifera.” Current Biology 28, no. 16: 2536–2543.e5. 10.1016/j.cub.2018.06.027. 30078568 PMC6783311

[mbo370368-bib-0081] Yasuhara, M. , D. P. Tittensor , H. Hillebrand , and B. Worm . 2017. “Combining Marine Macroecology and Palaeoecology in Understanding Biodiversity: Microfossils as a Model: Marine Macroecology–Palaeoecology Integration.” Biological Reviews 92, no. 1: 199–215. 10.1111/brv.12223. 26420174

[mbo370368-bib-0082] Záhonová, K. , G. Lax , S. D. Sinha , et al. 2021. “Single‐Cell Genomics Unveils a Canonical Origin of the Diverse Mitochondrial Genomes of Euglenozoans.” BMC Biology 19, no. 1: 103. 10.1186/s12915-021-01035-y. 34001130 PMC8130358

[mbo370368-bib-0083] Zardoya, R. 2020. “Recent Advances in Understanding Mitochondrial Genome Diversity.” F1000Research 9: 270. 10.12688/f1000research.21490.1. PMC7194472 32399193

[mbo370368-bib-0084] Žárský, V. , A. Karnkowska , V. Boscaro , et al. 2023. “Contrasting Outcomes of Genome Reduction in Mikrocytids and Microsporidians.” BMC Biology 21, no. 1: 137. 10.1186/s12915-023-01635-w. 37280585 PMC10245619

[mbo370368-bib-0085] Zimorski, V. , C. Ku , W. F. Martin , and S. B. Gould . 2014. “Endosymbiotic Theory for Organelle Origins.” Current Opinion in Microbiology 22: 38–48. 10.1016/j.mib.2014.09.008. 25306530

