# Supplementary material for: Mitochondrial Genome Reduction and Accelerated Evolution in Planktonic Foraminiferans
Source: Microbiologyopen. 2026 Jul 30;15(4):e70368. doi: 10.1002/mbo3.70368 (PMC13421374; doi:10.1002/mbo3.70368)
Supplement: Supplementary file 1 — Figure S1: Mitochondrial COX1 protein phylogeny of Foraminifera, including three particles from Green Island (GI) seawater. Figure S2: The frequency of repetitive sequences in the mitogenomes of globothalmid foraminiferans. Figure S3: Individual mitochondrial gene trees of Rhizaria. Table S1: Complete or nearly complete rhizarian mitogenome sequences analyzed in this study. Table S2: Taxonomic distribution of contigs (%) in each planktonic foraminiferan (Globigerinidae) particle SAG. [file MBO3-15-e70368-s001.docx]

**Supplementary Tables and Figures**

**Supplementary Table 1. Complete or nearly complete rhizarian mitogenome sequences analyzed in this study.**

| Species | Accession ID/References | Higher-level taxa | Note |
| --- | --- | --- | --- |
| *Calcarina hispida* | OP965950.1 | Foraminifera (Globothalamea) |  |
| *Neorotalia gaimardi* | OP965949.1 | Foraminifera (Globothalamea) |  |
| *Spiculammina delicata* | PV138223 | Foraminifera (Monothalamea) | Added during revision |
| *Psammina aff. limbata* | PV138224 | Foraminifera (Monothalamea) | Added during revision |
| *Acanthometra* sp*.* | Macher, J.-N et al., 2023 | Radiolaria | Nearly complete |
| *Lithomelissa* sp. | Macher, J.-N et al., 2023 | Radiolaria | Nearly complete |
| *Polymyxa betae* | NC_059071.1 | Endomyxa |  |
| *Spongospora subterranea* | NC_034004.1 | Endomyxa |  |
| *Lotharella oceanica* | NC_029731.1 | Cercozoa |  |
| *Paracercomonas marina* | KP165385 | Cercozoa |  |
| *Bigelowiella natans* | HQ840955 | Cercozoa | Nearly complete |

**Supplementary Table 2. Taxonomic distribution of contigs (%) in each planktonic foraminiferan (Globigerinidae) particle SAG**

| Particle | GI-A | GI-B | GI-C | GI-D |
| --- | --- | --- | --- | --- |
| Prokaryotic contigs | 97.0% | 84.7% | 74.6% | 68.9% |
| Eukaryotic contigs | 2.7% | 15.1% | 22.5% | 28.9% |
| Unclassified contigs | 0.3% | 0.2% | 2.9% | 2.2% |

**
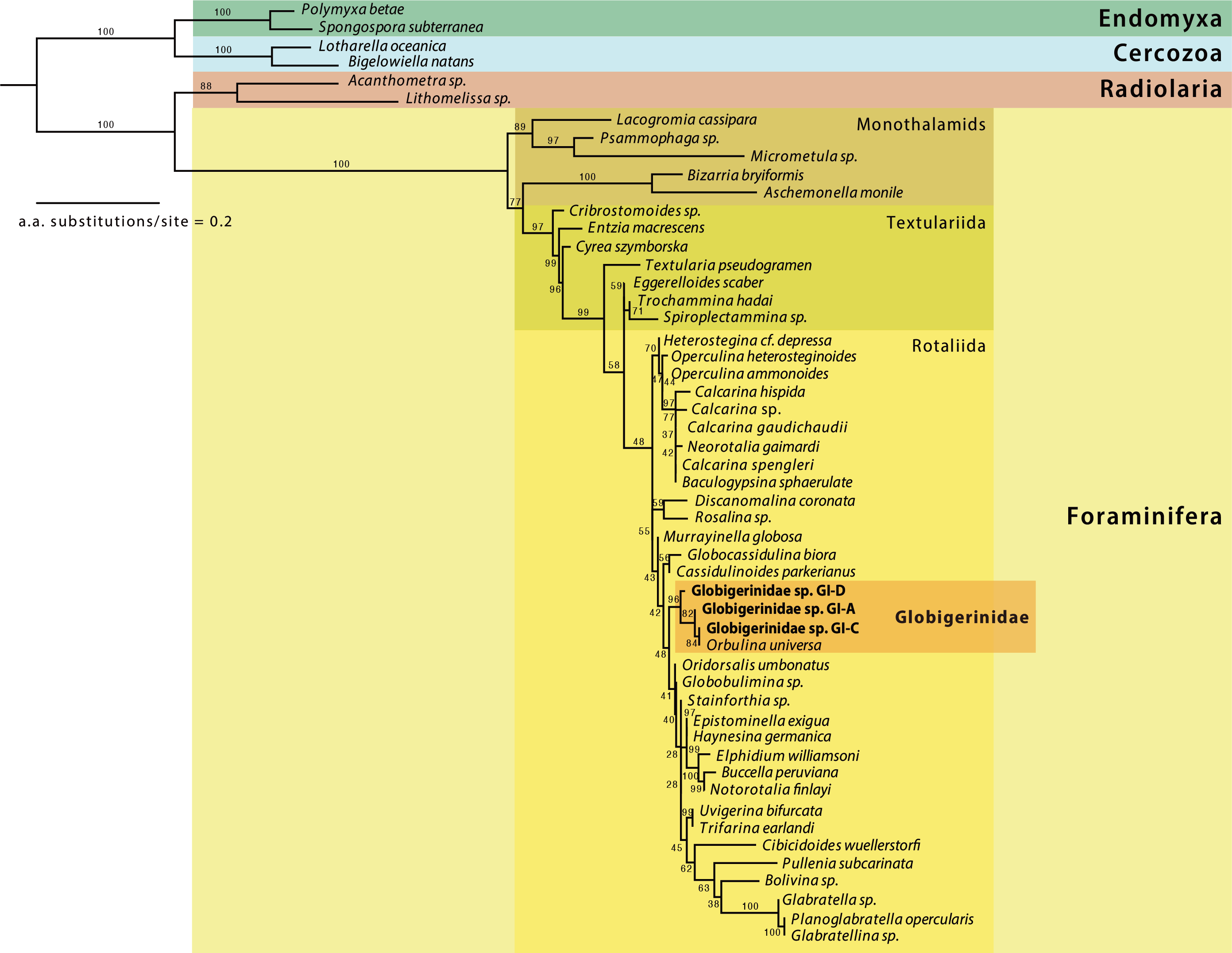
**

**Supplementary Figure 1. Mitochondrial COX1 protein phylogeny of Foraminifera, including three particles from Green Island (GI) seawater.** The maximum likelihood tree was reconstructed from COX1 protein sequences of 52 rhizarians (1240 aligned amino acid sites) using IQ-TREE, with the mtZOA model. Planktonic foraminiferans GI-A, GI-C, and GI-D are clustered with *Orbulina universa* (Globigerinidae, Rotaliida). Bootstrap support values (%, based on 1000 replicates) are shown at the branches.


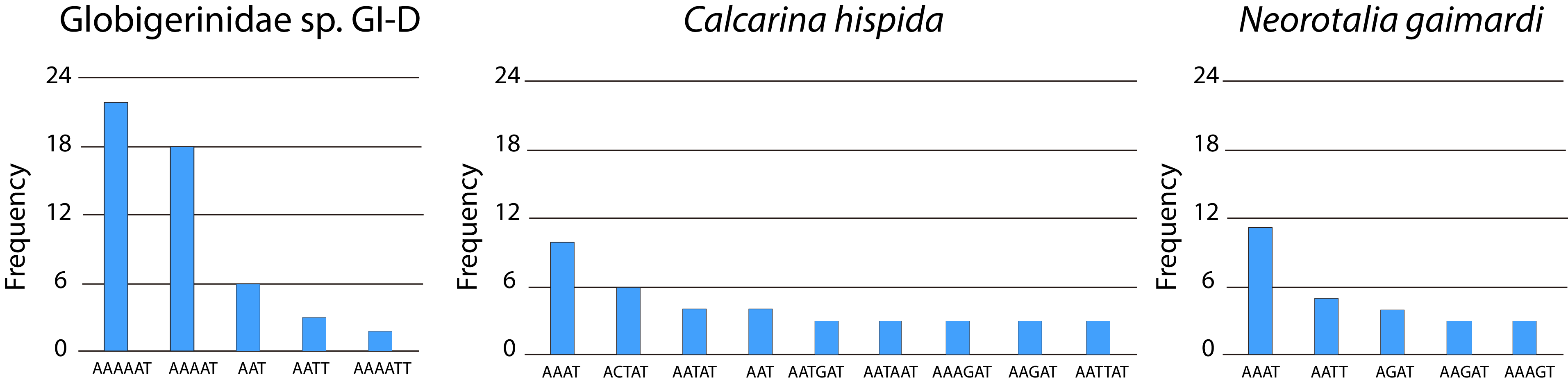


**Supplementary Figure 2. The frequency of repetitive sequences in the mitogenomes of globothalmid foraminiferans.** The bar chart shows the top five frequent repetitive DNA sequences in Globigerinidae sp. GI-D, *C. hispida*, and *N. gaimardi*.


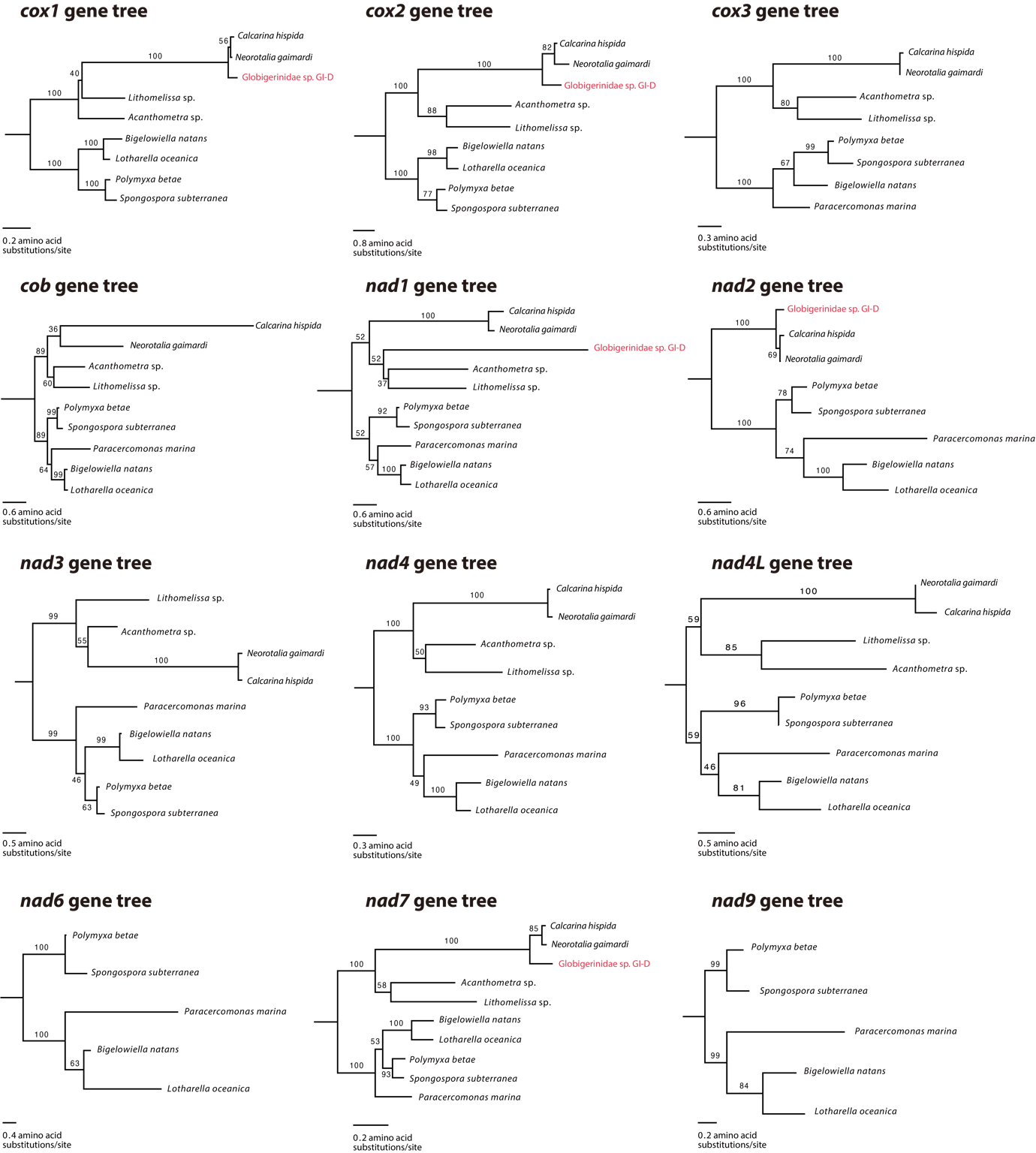


**Supplementary Figure 3. Individual mitochondrial gene trees of Rhizaria.** Mitochondrial *cox1*, *cox2*, *cob*, *nad1*, *nad3*, *nad4*, *nad4L*, and *nad7* gene trees reveal an accelerated evolutionary rate in Foraminifera. The trees were constructed based on protein sequences, and the bootstrap support values (%, based on 1000 replicates) are shown at the branches.
